# Ultrasound Images That Speak: Assessing the Therapeutic Decision in the Emergency Department Regarding the Risk–Benefit Ratio of Systemic Thrombolysis in Intermediate-High-Risk Pulmonary Embolism—A Case Report

**DOI:** 10.3390/diagnostics16010048

**Published:** 2025-12-23

**Authors:** Adela Golea, Raluca Mihaela Tat, Carina Adam, Sonia Luka, Mirela Anca Stoia, Ștefan Cristian Vesa

**Affiliations:** 1 Cluj-Napoca County Emergency Clinical Hospital, 3-5 Clinicilor Street, 400347 Cluj-Napoca, Romania; adela.golea@umfcluj.ro (A.G.); carinaadam17@gmail.com (C.A.); sonia.luka@umfcluj.ro (S.L.); 2 Department 6 Surgery, Emergency Medicine Discipline, “Iuliu-Hatieganu” University of Medicine and Pharmacy, 8 Victor Babes Street, 400347 Cluj-Napoca, Romania; 3 Department 4 Internal Medicine, Cardiology Medicine Discipline, “Iuliu-Hatieganu” University of Medicine and Pharmacy, 8 Victor Babes Street, 400347 Cluj-Napoca, Romania; 4 Cluj-Napoca Clinical Hospital of Infectious Diseases, 23 Iuliu Moldovan Street, 400000 Cluj-Napoca, Romania; stefan.vesa@umfcluj.ro; 5 Department 1 Functional Sciences, Discipline of Pharmacology, Toxicology and Clinical Pharmacology, Faculty of Medicine, “Iuliu-Hatieganu” University of Medicine and Pharmacy, 23 Marinescu Street, 400337 Cluj-Napoca, Romania

**Keywords:** pulmonary embolism, massive thrombotic burden, right atrial thrombus, systemic thrombolysis, Emergency Department, POCUS-TTE

## Abstract

**Background**: The management of acute pulmonary embolism (PE) in the Emergency Department (ED) remains challenging, particularly in hemodynamically and respiratory stable patients with minimal symptoms. Diagnostic and therapeutic difficulties are further compounded when the condition is complicated by a mobile right atrial (RA) thrombus, representing an extreme-risk phenotype. **Case Presentation:** We report the case of a 65-year-old male with a single known venous thromboembolism risk factor-chronic venous insufficiency-who presented to the ED following a transient episode of severe dyspnea at home. On admission, he was hemodynamically and respiratory stable, without the need for oxygen supplementation. Arterial blood gas analysis revealed a metabolically compensated acidosis with elevated lactate, while cardiac biomarkers were moderately increased. Emergency point-of-care transthoracic echocardiography (POCUS-TTE) demonstrated severe right ventricular (RV) dysfunction and a large, mobile intracardiac thrombus prolapsing through the tricuspid valve. Computed Tomography Pulmonary Angiography confirmed pulmonary embolism and revealed a massive and extensive bilateral thrombotic burden (Qanadli score 32 points). Given the extreme risk for fatal embolization, immediate full-dose systemic thrombolysis with Alteplase (100 mg over 2 h) was initiated in the ED. Thrombolysis was completed without hemorrhagic complications. Follow-up POCUS-TTE at 2 h showed complete resolution of the intracardiac thrombus and significant improvement of RV function (RV/RA gradient reduced from 40 mmHg to 28 mmHg). **Conclusions**: This case highlights the effectiveness and safety of early systemic thrombolysis guided by ED POCUS-TTE in PE with a massive thrombotic burden, complicated by a mobile intracardiac thrombus, even in the absence of shock. Such prompt intervention may reduce mortality risk in intermediate-to-high-risk PE subsets, despite limited guidance in current clinical recommendations.

## 1. Introduction

Acute pulmonary embolism (PE) remains a major cause of cardiovascular mortality worldwide [1,2]. Risk stratification is the cornerstone of management, with current guidelines recommending immediate reperfusion therapy only for massive PE, defined by the presence of systemic hypotension [3,4,5]. However, the diagnosis of PE may represent a significant clinical challenge, particularly in patients who are hemodynamically stable and present with minimal or nonspecific symptoms. The detection of a mobile thrombus traversing the right heart—often a serpentine clot referred to as a “thrombus in transit”—is universally recognized as a powerful, independent predictor of adverse outcomes, with reported mortality rates approaching 80% when left untreated [6,7,8]. Despite this, current international guidelines do not advocate for routine systemic thrombolysis in intermediate-high-risk PE, leaving management decisions in such scenarios highly individualized and clinically demanding [3].

This case aims to illustrate the diagnostic complexity and therapeutic challenges encountered in intermediate-high-risk PE complicated by a large, mobile intracardiac thrombus in a paucisymptomatic patient.

In this report, we present the clinical situation of a male patient who experienced a single transient episode of severe dyspnea, with paraclinical and laboratory findings revealing lactic metabolic acidosis and ischemic hepatorenal impairment. The values of cardiac biomarkers were moderately elevated, but nonspecific for the life-threatening conditions initially considered in the differential diagnosis, such as myocardial infarction, PE, or aortic dissection. Point-of-care-ultrasound transthoracic echocardiography (POCUS-TTE) was highly suggestive of PE, revealing a large, mobile intracardiac thrombus prolapsing through the tricuspid valve, which prompted further confirmatory imaging and underscored the challenges of therapeutic management.

## 2. Materials and Methods

All clinical data, images, and video materials are presented in accordance with institutional ethical standards, the Declaration of Helsinki, and the International Ethical Guidelines for Health-related Research Involving Humans. All information has been managed in compliance with the European Union General Data Protection Regulation, ensuring full protection of patient identity and privacy.

### 2.1. Point-of-Care Blood Analysis

Point-of-care blood testing was performed to evaluate cardiac biomarkers and arterial blood gases. Peripheral venous blood was collected in heparinized syringes for the determination of cardiac biomarkers, including high-sensitivity cardiac troponin I (hs-cTnI), N-terminal pro-B-type Natriuretic Peptide (NT-proBNP), and D-dimer, using the PATHFAST^®^ analyzer (Mitsubishi Chemical Medience Corporation, Tokyo, Japan). Arterial blood samples were obtained in heparinized syringes for blood gas analysis, which was carried out using the Stat Profile PRIME^®^ analyzer (Nova Biomedical, Waltham, MA, USA). All measurements were performed in the Emergency Department (ED) of the Cluj-Napoca County Emergency Clinical University Hospital, Romania, according to the manufacturers’ standard operating procedures and institutional laboratory quality control protocols.

The complete blood count, serum biochemistry, and coagulation profile were processed at the Central Laboratory of the Cluj-Napoca County Emergency Clinical Hospital, Cluj-Napoca, Romania, using a Mindray BC-6200 hematology analyzer (Shenzhen Mindray Bio-Medical Electronics Co., Ltd., Shenzhen, China), a Beckman Coulter AU680 biochemistry analyzer (Beckman Coulter, Brea, CA, USA), and an ACL TOP 550 coagulation analyzer (Instrumentation Laboratory, Bedford, MA, USA), respectively.

### 2.2. Point-of-Care-Ultrasound Transthoracic Echocardiography (POCUS—TTE)

TTE was performed as a POCUS examination using a Versana Active ultrasound system (General Electric Healthcare, Chicago, IL, USA). The examination included standard parasternal long and short-axis, apical four-chamber, and subcostal views. The assessment focused on right ventricular (RV) size and function, estimation of systolic pulmonary artery pressure, and the detection of intracardiac thrombi or other potential causes of acute right heart strain.

### 2.3. Computed Tomography Pulmonary Angiography (CTPA)

CTPA was performed using a General Electric Healthcare 8-slice IMA 660 scanner (Milwaukee, WI, USA), with intravenous administration of Omnipaque™ 350 contrast agent (GE General Electric Healthcare, Chicago, IL, USA).

## 3. Case Presentation

### 3.1. Clinical Summary

A 65-year-old male called emergency medical services (at 10:45) for progressive exertional dyspnea and fatigue over the previous four days, culminating on the day of admission in a transient episode of severe dyspnea, chest pain, peripheral cyanosis, and mottled, cold skin, with an oxygen saturation (SpO_2_) of 76%. Upon arrival at the Emergency Department (at 12:02), his vital signs were within normal limits: SpO_2_ 98% on room air, heart rate 108 bpm (sinus tachycardia), and blood pressure 139/104 mmHg. His past medical history included an ischemic stroke without residual deficits (2022), chronic venous insufficiency with prior varicose vein surgery (at the age of 35), and no other known cardiovascular or respiratory disease. Chronic home medication consisted of Aspirin (Aspenter^®^), Atorvastatin (Atoris^®^), Pentoxifylline, and Diosmin-Hesperidin (Detralex^®^). Comprehensive physical examination across all systems revealed no abnormal clinical findings at presentation.

### 3.2. Diagnostic Findings

The electrocardiogram performed upon admission showed sinus rhythm at 100 bpm, a biphasic P wave in lead V_1_, an S_1_Q_3_ pattern, prominent P waves in leads II and III (“P pulmonale”), and signs of acute RV overload with anterior T-wave inversions (at 12:20).

Arterial blood gas analysis indicate a mixed acid–base disorder, consisting of lactic metabolic acidosis and concomitant respiratory alkalosis, with a near-normal pH reflecting partial compensation between the two opposing mechanisms. Overall oxygenation was preserved at the time of sampling (PaO_2_/FiO_2_ = 415); however, a mildly increased alveolar–arterial oxygen gradient (A–a DO_2_) suggested a ventilation–perfusion mismatch, consistent with a suspected pulmonary embolism (at 12:22).

Complete blood count revealed leukocytosis with neutrophilia, and a high-sensitivity C-reactive protein (hs-CRP) exceeding 30 mg/L. Biochemical testing revealed hepatic cytolysis with liver enzyme levels approximately twice the upper normal limit, incipient renal impairment associated with reduced glomerular filtration rate, and elevated lactate dehydrogenase, findings suggestive of systemic circulatory insufficiency with impaired tissue oxygenation (between 12:22 and 13:30). The laboratory parameters obtained in the ED are presented in Table 1.

Cardiac biomarkers revealed a markedly positive D-dimer, which, while nonspecific, could not rule out active thrombosis and raised suspicion for venous thromboembolism or other acute prothrombotic states. Myocardial injury was indicated by elevated hs-cTnI, a finding that may reflect RV strain secondary to PE, but could also occur in acute coronary syndromes or myocarditis. The severity of ventricular wall stress was further supported by a markedly increased NT-proBNP level, consistent with acute ventricular overload and pressure elevation (at 12:38) (Table 2).

The assessment of clinical probability for pulmonary embolism using standardized risk scores revealed discordant results. Wells’ criteria, classifying the patient in the low-risk group, corresponding to an estimated 1.3% probability of PE in an Emergency Department population. In contrast, the revised Geneva score classified the patient into the intermediate-probability group, corresponding to an estimated 20–30% likelihood of PE (Table 2).

**Figure 1:**
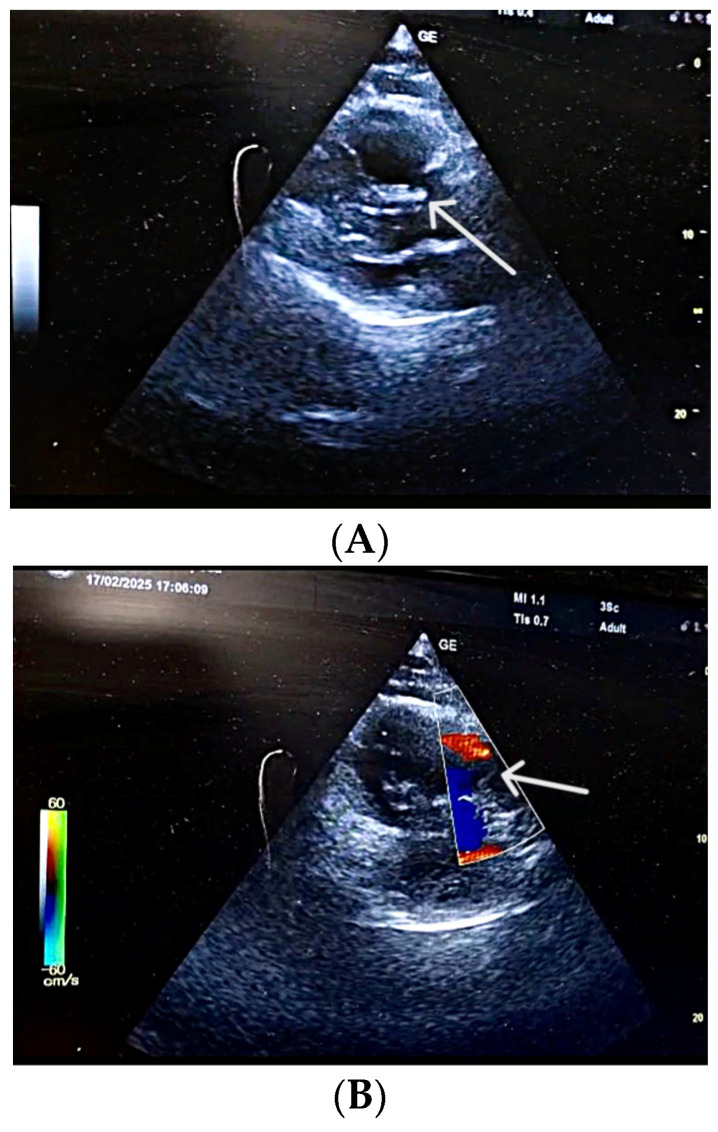
(**A**) **POCUS-TTE performed pre-thrombolysis, showing a markedly dilated right ventricular cavity.** Parasternal long-axis view showing acute dilatation of the right ventricle and pulmonary annulus, resulting in incomplete coaptation of the pulmonary valve cusps secondary to an abrupt increase in pulmonary artery pressure (white arrow). (**B**) **POCUS-TTE performed pre-thrombolysis, showing sudden right ventricular pressure overload.** Parasternal short-axis view showing the main pulmonary artery trunk with functional pulmonary regurgitation (white arrow).

**Figure 2:**
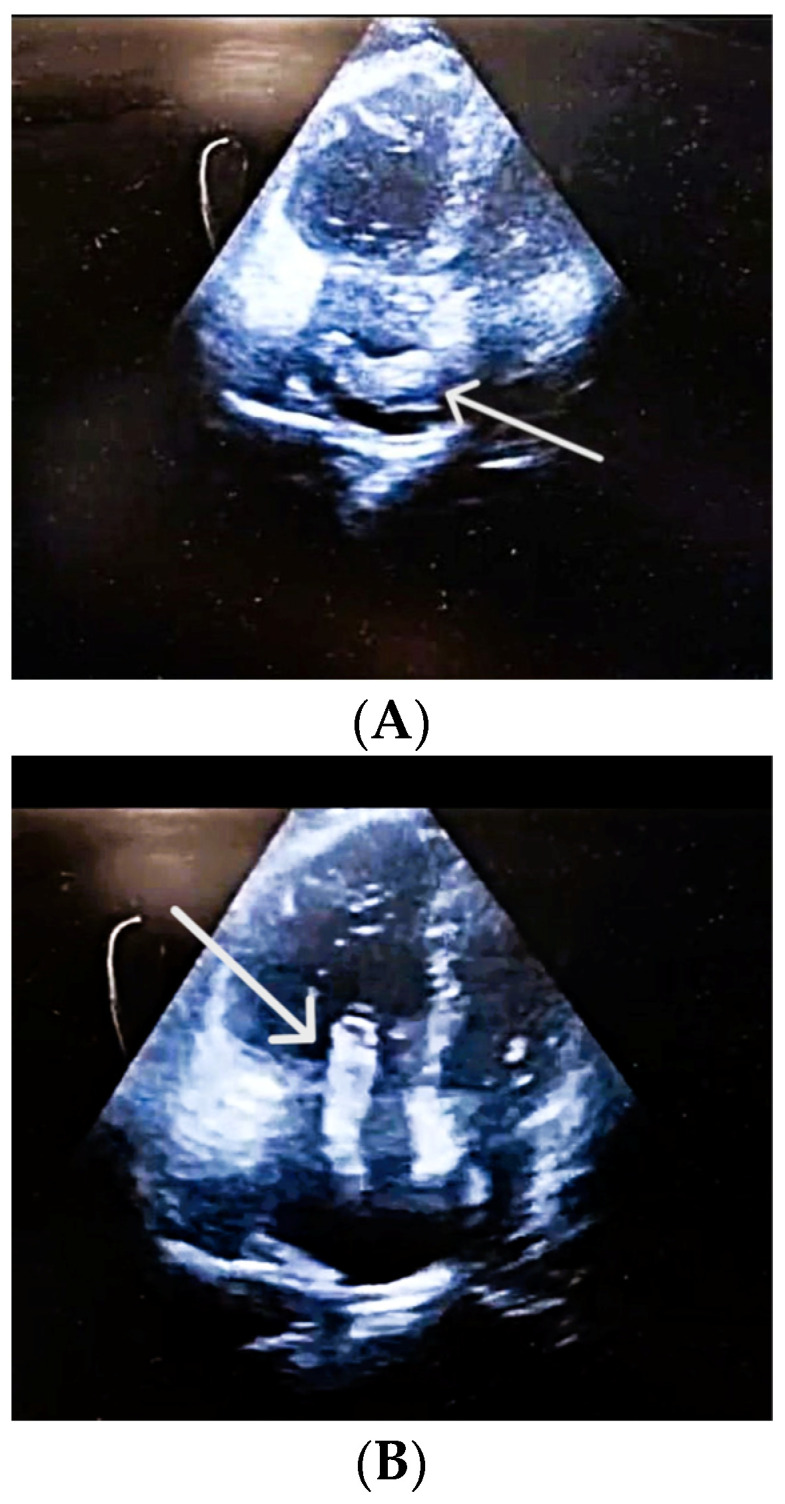
(**A**) **POCUS-TTE performed pre-thrombolysis, showing a thrombus in transit.** Apical four-chamber view demonstrating a large, mobile thrombus (in a horizontal position) within the right atrium (white arrow). (**B**) **POCUS-TTE performed pre-thrombolysis, showing a thrombus in transit.** Apical four-chamber view showing a large, serpentine, free-floating thrombus (white arrow) within the right atrium, seen prolapsing toward the tricuspid valve. Right ventricular cavity is larger than the left ventricular cavity RV/LV > 1.

In the absence of any anamnestic or clinical evidence suggestive of pulmonary or myocardial infection (rapid influenza A/B and COVID-19 tests were also negative), acute myocardial infarction, or aortic dissection, and considering the laboratory findings, the clinical suspicion of PE became increasingly prominent, prompting the immediate performance of POCUS-TTE. Performed at 14:00, the POCUS-TTE examination confirmed the physiological impact of acute RV strain (Figure 1A,B), demonstrating severe RV dilation (46 mm below the tricuspid annulus), markedly reduced systolic function (tricuspid annular plane systolic excursion, TAPSE = 12 mm), and paradoxical interventricular septal motion resulting in a “D-shaped” left ventricle (LV)—findings consistent with acute RV pressure overload [RV-right atrium (RA) pressure gradient 40 mmHg]. Crucially, a large, highly mobile, serpentine thrombus was visualized within the RA, with its free-floating end prolapsing toward the tricuspid valve orifice (Figure 2A,B). The concomitant presence of asymptomatic left popliteal deep vein thrombosis (DVT) was also documented, with the thrombus showing a non-adherent proximal end and an unstable, mobile appearance, further supporting the diagnosis of PE (Table 3).

**Figure 3:**
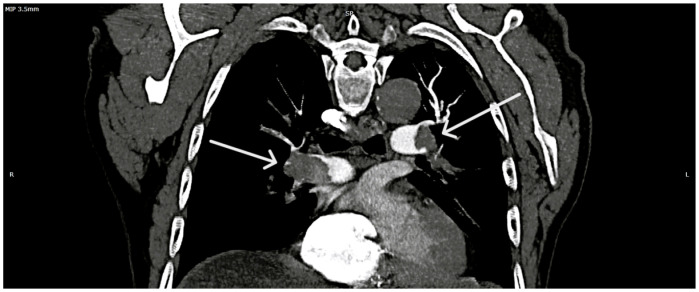
**Massive and extensive bilateral pulmonary thrombotic burden.** Axial CTPA view illustrating extensive, multi-territory thromboembolic involvement (white arrows) of the main and lobar pulmonary arteries, consistent with a burden equivalent to massive PE. **Legend:** R—right side; L—left side (of the image).

Given that the therapeutic decision depended on the presence or absence of PE and on the extent of vascular involvement secondary to thrombosis, and taking into account the patient’s hemodynamic stability, a pulmonary CTPA was performed (at 17:08 p.m.). CTPA images revealed a massive and extensive bilateral pulmonary thrombotic burden, confirming the diagnosis and showing a severity far greater than initially anticipated by the medical team (Figure 3). The calculation of the Qanadli score, used to assess thrombotic burden in PE based on CTPA images, revealed a total of 32 points out of a maximum of 40. This score classifies the patient as having the equivalent of massive PE, a category associated with a high risk of hemodynamic instability and mortality, with an obstruction degree exceeding 80%.

### 3.3. Therapeutic Management and Immediate ED Outcome

In light of the extreme risk associated with the mobile intracardiac thrombus and the high thrombotic burden of PE, the decision was made to bypass delayed interventions (surgical or catheter-based embolectomy) and to initiate rapid full-dose systemic thrombolysis (Alteplase 100 mg administered over 2 h, the patient weighing more than 65 kg) directly in the ED, under continuous respiratory and hemodynamic monitoring (at 18:00 p.m.—four hours after performing the POCUS-TTE, and less than one hour after the computed tomography was completed).

Before administering systemic thrombolysis, a focused bleeding-risk evaluation was performed according to guideline-defined absolute and relative contraindications. The patient had no history of intracranial hemorrhage, no recent ischemic stroke, no major surgery, trauma, or active bleeding, and laboratory testing showed normal platelet count and no coagulopathy. His prior minor ischemic stroke (2022) was sufficiently distant in time and fully recovered, therefore not representing a contraindication. The patient was not receiving anticoagulant therapy, only low-dose aspirin as an antiplatelet agent, which does not contraindicate fibrinolysis. Given the absence of bleeding-risk factors and the extreme-risk anatomical context (a large, mobile RA thrombus with severe RV dysfunction) the risk-benefit analysis clearly favored urgent full-dose systemic thrombolysis.

**Figure 4:**
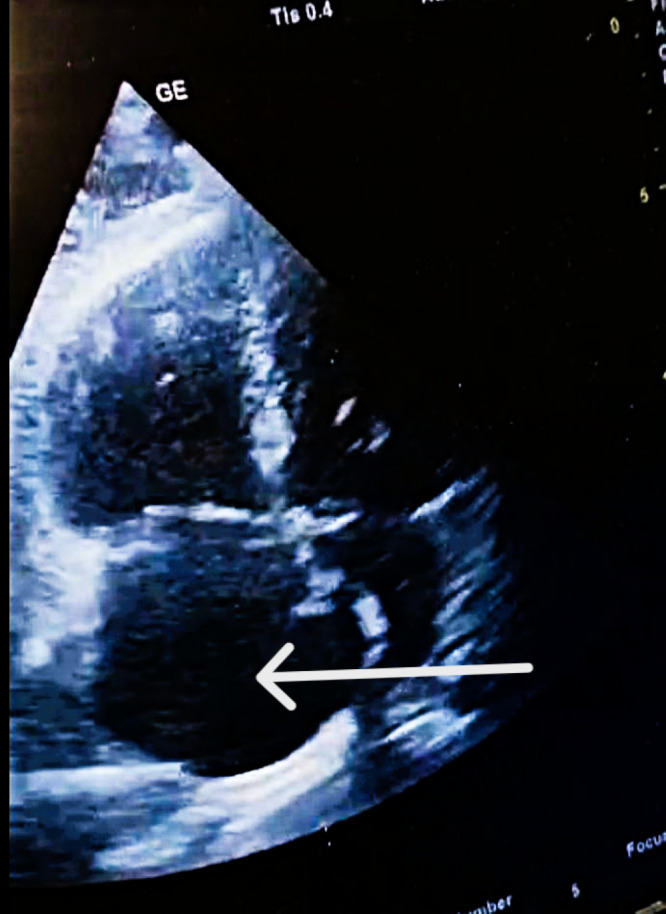
**POCUS**—TTE post-thrombolysis. **Complete resolution of intracardiac thrombus following Emergency Department thrombolysis.** Follow-up POCUS-TTE (apical four-chamber view) performed two hours after Alteplase infusion demonstrates complete clearance of the right atrium, with no residual mobile thrombus visible (white arrow).

Safety surveillance during and immediately after the infusion confirmed the absence of major bleeding complications, with specific exclusion of intracranial hemorrhage. A follow-up POCUS-TTE performed two hours after thrombolysis initiation confirmed the primary therapeutic endpoint—complete resolution of the mobile RA thrombus. Additionally, there was evidence of partial reversal of RV pressure overload, demonstrated by a reduction in the RV—RA gradient from 40 mmHg to 28 mmHg (at 20:05) (Figure 4).

### 3.4. Cardiological Clinical Course During Hospital Stay

The patient was admitted to the Interventional Cardiology Department, with a total hospitalization duration of 7 days, during which several cardiology-driven clinical considerations guided the subsequent therapeutic decisions.

In the absence of solid clinical or laboratory indicators suggestive of a septic state, procalcitonin measurement was not performed at admission (it was measured on day 3, and the result was within the normal range) (Table S1, Supplementary Materials). Considering the inflammatory status, although no definite infectious source was identified, but taking into account the patient’s severe acute pathology, the patient received oral antibiotic therapy with Amoxicillin–Clavulanic acid (Amoxiplus^®^) 1000/200 mg every 12 h, starting from the first day of admission and continued until discharge. During the evolution, four days after admission, inflammatory markers such as leukocytes and hs-CRP showed a decreasing trend (Table S1, Supplimentary Materials). The chest X-ray (the second one) performed in the cardiology ward did not reveal any infectious process. Repeated testing for influenza and COVID-19 remained negative throughout the hospitalization.

After thrombolysis, the patient received intravenous anticoagulant therapy with unfractionated Heparin, administered as a continuous infusion at a dose of 12 IU/kg/h, initiated 12 h after completion of thrombolysis, with aPTT values monitored every 6 h. At 24 h post-thrombolysis, anticoagulation was switched to enoxaparin 4000 IU (Clexane^®^) every 12 h for 2 days, followed by oral anticoagulation with the Factor Xa inhibitor rivaroxaban (Xarelto^®^) at a dose of 15 mg every 12 h starting on day 4 (laboratory tests confirmed normalization of renal function by day 4 of hospitalization—Table S1, Supplimentary Materials).

The patient had a known history of arterial hypertension, smoking, dyslipidemia, prior ischemic stroke without residual deficits, and surgically treated varicose veins. His chronic home medication included antiplatelet therapy (acetylsalicylic acid, Aspenter^®^ 75 mg/day), phlebotropic therapy (diosmin 450 mg and hesperidin 50 mg, Detralex^®^ 500 mg every 12 h), a peripheral vasodilator (pentoxifylline 400 mg every 12 h), and statin therapy (atorvastatin, Atoris^®^ 40 mg/day), without any antihypertensive medication. The shock index (heart rate/systolic blood pressure ratio) upon arrival in the ED was 0.77, with a mean arterial pressure of 115 mmHg, indicating no signs of shock at presentation. In this clinical context, the initially elevated diastolic blood pressure values—correlated with subsequent POCUS-TTE findings and the overall clinical picture at admission and throughout hospitalization—were considered to have a multifactorial origin, including pre-existing arterial hypertension, acute stress with sympathetic activation, and probably early compensatory vasoconstrictive mechanisms. Correction of hypertension required the administration of diuretics (Furosemide^®^, 40 mg every 12 h). During hospitalization, the patient also continued statin therapy (atorvastatin, Tarvacard^®^ 20 mg every 12 h).

Dynamic transthoracic echocardiography performed prior to discharge showed preserved LV systolic function, signs of improved RV loading conditions, and no evidence of new intracardiac thrombi (Supplimentary Materials).

Rivaroxaban was continued at discharge (7 days after admission), prescribed at 15 mg twice daily for 2 weeks, followed by 20 mg once daily in the morning for a total duration of 6 months. The patient was also referred for comprehensive hematological evaluation to investigate possible underlying thrombophilia or other prothrombotic conditions. Unfortunately, the patient did not attend the scheduled 1-month cardiology follow-up appointment; therefore, no additional imaging (echocardiography or CTPA) and no long-term functional assessment could be obtained. Consequently, chronic thromboembolic pulmonary hypertension could not be formally excluded.

## 4. Discussion

The management of acute PE remains a major clinical challenge, particularly when complicated by a mobile RA thrombus (thrombus in transit), a condition associated with an exceptionally high risk of mortality if left untreated [3,6]. The presence of such a thrombus indicates ongoing embolization and represents an extreme-risk anatomical phenotype, even in patients who initially present as hemodynamically stable. Reported mortality rates for untreated right-heart thrombi range from 27% to over 80%, underscoring the urgency of immediate recognition and intervention [6,9].

In the present case, the diagnostic process was notably complex due to the atypical and transient clinical presentation, which consisted of nonspecific symptoms such as progressive exertional dyspnea and fatigue, without overt hemodynamic or respiratory failure. Such paucisymptomatic profiles are not uncommon in submassive or intermediate-high-risk PE and can easily lead to a superficial diagnostic approach or initial misdiagnosis (e.g., pneumonia, heart failure, or anxiety-related dyspnea), delaying the initiation of potentially life-saving therapy. The combination of elevated but nonspecific biomarkers (hs-cTnI, NT-proBNP, D-dimers) and subtle clinical findings emphasized the importance of maintaining a high index of suspicion and proceeding to early imaging.

In this context, POCUS-TTE proved to be a pivotal diagnostic tool, providing immediate, bedside evidence of severe RV dilation and dysfunction, along with the visualization of a large, highly mobile intracardiac thrombus prolapsing through the tricuspid valve. This single finding was highly suggestive of an active embolic process, prompting urgent CTPA, which confirmed a massive bilateral thromboembolic burden. Emergency POCUS—TTE detection of a mobile RA thrombus is among the most specific and prognostically significant findings in acute PE, as it identifies an ongoing source of emboli and predicts imminent hemodynamic collapse. Several studies have demonstrated that right-heart thrombi are independently associated with increased mortality, regardless of initial blood pressure values [6,7,8,9]. Therefore, POCUS-TTE serves not only as a diagnostic but also as a prognostic and decision-shaping tool, especially in the ED setting, where time-sensitive decisions are crucial [9,10,11].

Although the patient exhibited a massive angiographic clot burden on CTPA, his hemodynamic profile did not meet the ESC guideline definition of high-risk (massive) PE, as systemic hypotension or shock were absent. According to the 2019 ESC criteria, the patient fulfilled the requirements for intermediate-high-risk PE, based on RV dilation and dysfunction demonstrated on POCUS-TTE, together with elevated cardiac biomarkers (hs-cTnI and NT-proBNP) (3). This case therefore highlights the important distinction between anatomical severity—reflected by extensive thrombotic obstruction—and guideline-based clinical risk categories, which are determined primarily by hemodynamic status and evidence of RV strain. Recognizing this conceptual separation is essential when evaluating therapeutic options, as a massive clot burden alone does not redefine the formal risk class but may justify individualized, escalated treatment strategies in anatomically extreme-risk scenarios.

Our therapeutic approach diverged from the strategies employed in major clinical trials evaluating thrombolytic therapy in PE. The MOPETT trial [12] investigated half-dose Alteplase (50 mg) in moderate PE and demonstrated a significant reduction in pulmonary hypertension and recurrence rates, without an increased risk of major bleeding. Conversely, the PEITHO trial [13] used full-dose Tenecteplase in hemodynamically stable patients with RV dysfunction and elevated cardiac biomarkers, showing fewer episodes of hemodynamic decompensation but a higher rate of major hemorrhage, including intracranial bleeding.

However, neither of these trials specifically addressed patients with mobile intracardiac thrombi, where the embolic risk is dynamic and potentially catastrophic. In our case, the presence of a large, serpentine, free-floating RA thrombus, together with an extensive and massive pulmonary thrombotic burden, represented an extreme-risk anatomical scenario, justifying immediate reperfusion therapy despite preserved hemodynamics. Consequently, we administered full-dose systemic thrombolysis (Alteplase 100 mg over 2 h) directly in the ED, under continuous monitoring.

Our decision contrasts with the dosing regimens used in several recent observational studies, including the work by Ömer Selim Unat et al. [14], where half-dose Alteplase (50 mg) was employed in intermediate-high–risk PE, yielding favorable outcomes without major bleeding complications. Nonetheless, data remain limited regarding full-dose thrombolysis in hemodynamically stable patients with an intracardiac thrombus.

While thrombus-in-transit has been previously described in several case reports and small series, the individual components observed in our patient have not been documented together in a single report. What distinguishes the present case is the unique combination of features: a hemodynamically stable and paucisymptomatic presentation, an intermediate-high-risk PE accompanied by a large, mobile RA thrombus and a massive thrombotic burden, along with the use of full-dose systemic thrombolysis initiated directly in the ED. Most published cases involving intracardiac thrombi describe either hemodynamically unstable patients treated emergently with thrombolysis or surgical embolectomy [15,16], or stable but highly symptomatic patients who received reduced-dose fibrinolysis or delayed reperfusion [17]. In contrast, our patient remained normotensive, minimally symptomatic, and was treated with full-dose Alteplase administered immediately after multimodal imaging confirmation, resulting in complete thrombus resolution within two hours. This contextual comparison underscores how our case integrates several high-risk anatomical and procedural considerations seldom encountered simultaneously in published reports.

The clinical evolution strongly supports the safety and efficacy of this approach: the thrombus completely resolved within two hours post-thrombolysis, accompanied by significant improvement in RV function (RV—RA gradient decreased from 40 mmHg to 28 mmHg), without any hemorrhagic complications, including intracranial bleeding. These outcomes emphasize that, in selected patients, early full-dose systemic thrombolysis can be both feasible and effective when guided by clear anatomical and echocardiographic criteria rather than blood pressure alone.

Moreover, the feasibility of performing full-dose systemic thrombolysis directly in the ED is an important procedural message. This approach minimizes the critical door-to-needle time, prevents thrombus fragmentation and fatal embolization, and enables early hemodynamic stabilization. When performed under rigorous monitoring by an experienced emergency and cardiology team, the risk of major complications remains acceptably low.

Finally, the patient’s transition to oral anticoagulation with a Factor Xa inhibitor (Rivaroxaban) and referral for comprehensive hematological assessment ensured adequate secondary prevention and allowed for investigation of an underlying prothrombotic state, given the patient’s prior ischemic stroke and chronic venous insufficiency. Identifying such predispositions is critical for long-term management and recurrence prevention.

In summary, this case highlights several key learning points: 1. Nonspecific clinical presentations should not exclude the diagnosis of potentially life-threatening PE; 2. POCUS-TTE is an indispensable diagnostic and prognostic tool in the ED, capable of revealing RV dysfunction and mobile thrombi before hemodynamic collapse occurs; 3. Immediate, full-dose systemic thrombolysis can be justified in anatomically extreme-risk cases, even in the absence of hypotension, provided that the procedure is performed in a controlled, closely monitored environment.

### Limitation

This case report has several limitations that should be acknowledged. First, it describes the clinical course of a single patient, which inherently limits the generalizability of the findings and precludes any causal inference regarding the effectiveness or safety of full-dose systemic thrombolysis in hemodynamically stable patients with intermediate-high–risk PE and a mobile intracardiac thrombus. Second, this report reflects the experience of a single-center ED, and the therapeutic approach, particularly the decision to administer full-dose thrombolysis immediately in the ED, cannot be generalized to other settings or institutions that do not possess similar expertise, logistical capability, or rapid access to POCUS-TTE and interdisciplinary evaluation, even when patients appear clinically comparable. Moreover, we emphasize that the therapeutic decision in this case was the result of individualized clinical judgment, tailored to an extremely rare and high-risk anatomical scenario. In such exceptional circumstances, personalized management, aligned with current guidelines and supported by the treating team’s experience, is essential; however, it does not allow the extrapolation of these outcomes to the broader population of patients with PE. Finally, long-term clinical, paraclinical, and echocardiographic follow-up data are unavailable, as the patient did not attend the scheduled one-month cardiology evaluation, preventing further assessment of recurrence risk, functional recovery, or potential chronic thromboembolic complications.

## 5. Conclusions

A mobile RA thrombus complicating a PE with a massive thrombotic burden constitutes a true cardiovascular emergency, even in the absence of systemic hypotension or shock. This case demonstrates that POCUS-TTE, combined with prompt full-dose systemic thrombolysis initiated directly in the ED, can be both feasible and life-saving in selected patients with intermediate-high–risk PE and extreme-risk anatomical features. Although current guidelines do not formally endorse thrombolysis for intermediate-high–risk PE, this experience supports an individualized approach guided by real-time POCUS-TTE and anatomical findings rather than hemodynamic parameters alone. Early diagnosis through emergency POCUS-TTE, rapid initiation of reperfusion therapy, and comprehensive post-thrombolytic follow-up are essential components of optimal management aimed at improving survival and preventing recurrence.

## Figures and Tables

**Table 1:** Laboratory parameters at ED presentation.

Parameter Category	Test (U.M.)	ED Result	Reference Range
Hematology	WBC (10^9^/L)	15.44	4.00–10.00
	Neutrophils (10^9^/L)	11.27	2.00–7.00
	Lymphocytes (10^9^/L)	2.65	0.80–4.00
	Monocytes (10^9^/L)	1.48	0.12–1.20
	Eosinophils (10^9^/L)	0.01	0.02–0.50
	Basophils (10^9^/L)	0.03	0.00–0.10
	IMG (10^9^/L)	0.15	0.00–999.999
	RBC (10^12^/L)	4.51	3.50–5.50
	HBG (g/dL)	15.2	11–16
	HCT (%)	44.8	37–54
	MCV (fL)	99.3	80–100
	MHC (pg)	33.7	27–34
	PLT (10^9^/L)	207	100–300
	MPV (fL)	10.9	6.5–12
	PDW	16.8	16–17
	PCT (%)	0.226	0.108–0.282
Coagulation	aPTT (s)	24.0	23.5–36.5
	PT (s)	13.0	9–13
	Prothrombin index (%)	80	80–145%
	INR	1.16	0.79–1.16
ABG at FiO_2_ 21%	pH	7.437	7.35–7.45
	PaCO_2_ (mmHg)	20.9	35–45
	PaO_2_ (mmHg)	86.8	75–100
	SaO_2_ (%)	95	95–99
	O_2_Hb (%)	92.5	94–98
	COHb (%)	1.9	0.5–1.5
	HHb (%)	5.2	0.4–3
	O_2_Cap (mL/dL)	21.4	20
	A (mmHg)	118.5	Calculated alveolar formula
	O_2_Ct (mL/dL)	21	18–22
	A-aDO_2_ (mmHg)	31.7	<30 in older adults
	a/A	0.7	>0.75
	PaO_2_/FiO_2_	415.3	>300
	CcO_2_ (mL/dL)	21.4	Calculated formula
	CaO_2_ (mL/dL)	20.5	18–22
	HCO_3_^−^ (mmol/L)	14.1	22–26
	BE (mmol/L)	−10.7	−2 to +2
	Lactate (mmol/L)	1.8	<2.0
	Gap (mmol/L)	19.5	8–12
Liver Function	ALT (U/L)	80	<50
	AST (U/L)	113	<50
	BD (mg/dL)	0.43	<0.2
	TB (mg/dL)	1.67	0.3–1.2
Muscle Injury	CK (U/L)	86	<171
	CK-MB (U/L)	36	<24
Cellular Injury	LDH (U/L)	521	<248
Inflammation	hs-CRP mg/L	>30	<5
Renal Function	Creatinine (mg/dL)	1.70	0.67–1.17
	eGFR (mL/min/1.73 m^2^)	41.1	>60
	Urea (mg/dL)	87	17–43
Glycemic Status	Glucose (mg/dL)	176	74–106
Electrolytes	Na (mmol/L)	139	136–146
	K (mmol/L)	4.38	3.5–5.1
	Cl (mmol/L)	97	101–109
	Mg (mmol/L)	0.44	0.42–0.59

**Legend:** U.M.—unit of measure; ED—Emergency Department; WBC—white blood cells; Neutrophils—neutrophils; Lymphocytes—lymphocytes; Monocytes—monocytes; Eosinophils—eosinophils; Basophils—basophils; IMG—immature granulocytes; RBC—red blood cells; HBG—hemoglobin; HCT—hematocrit; MCV—mean corpuscular volume; MHC—mean hemoglobin content; PLT—platelets; MPV—mean platelet volume; PDW—platelet distribution width; PCT—plateletcrit; aPTT—activated partial thromboplastin time; PT—prothrombin time; INR—international normalized ratio; ABG—arterial blood gas; FiO_2_—fraction of inspired oxygen; PaCO_2_—arterial partial pressure of carbon dioxide; PaO_2_—arterial partial pressure of oxygen; SaO_2_—arterial oxygen saturation; O_2_Hb—oxyhemoglobin; COHb—carboxyhemoglobin; HHb—deoxyhemoglobin; O_2_Cap—oxygen capacity; A—alveolar oxygen pressure; O_2_Ct—oxygen content; A–aDO_2_—alveolar–arterial oxygen gradient; a/A—arterial-to-alveolar oxygen ratio; PaO_2_/FiO_2_—oxygenation ratio; CcO_2_—end-capillary oxygen content; CaO_2_—arterial oxygen content; HCO_3_^−^—bicarbonate; BE—base excess; Gap—anion gap; ALT—alanine aminotransferase; AST—aspartate aminotransferase; BD—direct bilirubin; TB—total bilirubin; CK—creatine kinase; CK-MB—creatine kinase MB isoenzyme; LDH—lactate dehydrogenase; hs-CRP—high-sensitivity C-reactive protein; eGFR—estimated glomerular filtration rate; Na—sodium; K—potassium; Cl—chloride; Mg—magnesium.

**Table 2:** Quantitative risk scores and key variables, POCUS-TTE findings and cardiac biomarkers, supporting the intermediate-high-risk classification.

**Wells Score—Parameters, Points and Risk Classification**			
Clinical signs and symptoms of DVT	No	1.5 points	Low risk group
PE is the first diagnosis OR equally likely	No		
Heart rate > 100 bpm	Yes		
Immobilization at least 3 days OR surgery in the previous 4 weeks	No		
Previous, objectively diagnosed PE or DVT	No		
Hemoptysis	No		
Malignancy or treatment within 6 months or palliative	No		
**Geneva Score—Parameters, Points and Risk Classification**			
Age > 65	Yes	6 points	Moderate risk group
Previous DVT or PE	No		
Surgery (under general anesthesia) or lower limb fracture in past month	No		
Active malignant condition Solid or hematologic malignant condition, currently active or considered cured < 1 year	No		
Unilateral lower limb pain	No		
Hemoptysis	No		
Heart rate	Yes		
Pain on lower limb palpation and unilateral edema	No		
**Simplified PESI—Parameters, Points and Risk Classification**			
Age, years > 80	No	0 points	Low risk group
History of cancer	No		
History of chronic cardiopulmonary disease	No		
Heart rate ≥ 110 bpm	No		
Systolic BP ≥ 100 mmHg	No		
O_2_ saturation > 90%	No		
**BOVA Score—Parameters, Points and Risk Classification**			
Systolic BP > 100 mmHg	Yes		
Elevated cardiac troponin	Yes	4 points	Intermediate risk
RV dysfunction	Yes		
Heart rate ≥ 110 bpm	No		
**Biomerkers—Parameters, Values and Risk Classification**			
D-dimer (µg/mL)	3.76	high	Suggestive of thrombosis
hs-cTnI (ng/mL)	119	high	Intermediate-high-risk
NT-proBNP (pg/mL)	15.136	high	Intermediate-high-risk
**POCUS-TTE—Parameters, Values and Risk Classification**			
RV/LV ratio	1.39	high	Intermediate-high-risk
TAPSE (mm)	12	low	Intermediate-high-risk

**Legend:** Wells score—clinical prediction score for pulmonary embolism; Geneva score—clinical prediction score for pulmonary embolism; PESI—Pulmonary Embolism Severity Index; DVT—deep vein thrombosis; PE—pulmonary embolism; bpm—beats per minute; BP—blood pressure; mmHg—millimeters of mercury; O_2_—oxygen; BOVA score—risk stratification score for pulmonary embolism; RV—right ventricle; LV—left ventricle; RV/LV ratio—right-to-left ventricular diameter ratio; D-dimer—fibrin degradation product; µg/mL—micrograms per milliliter; hs-cTnI—high-sensitivity cardiac troponin I; ng/mL—nanograms per milliliter; NT-proBNP—N-terminal pro-B-type natriuretic peptide; pg/mL—picograms per milliliter; POCUS-TTE—point-of-care transthoracic echocardiography; TAPSE—tricuspid annular plane systolic excursion.

**Table 3:** Key POCUS-TTE and Venous Ultrasound Measurements.

Parameter	Measurement	Interpretation
RV diameter	46 mm (below tricuspid/pulmonary annulus)	Marked RV dilation
LV diameter	33 mm	Normal size
RV/LV ratio	>1	Consistent with acute RV pressure overload
RA size	Dilated	Elevated right-sided pressures
Pulmonary trunk diameter	31 mm	Mild dilation; acute pulmonary hypertension
Tricuspid regurgitation, Vmax	3.2 m/s	Increased RV pressure
RV—RA gradient	40 mmHg	Significant RV overload
TAPSE	12 mm	Markedly reduced RV systolic function
IVC	19 mm, <50% collapse	Elevated RA pressure
Interventricular septum	Paradoxical motion	“D-shaped” LV; RV overload
Mobile RA thrombus	Large, serpentine, prolapsing through tricuspid	High embolic risk
Proximal DVT end	Lower third of superficial femoral vein	Non-adherent, unstable
Thrombus mobility	Free-floating, unstable	High embolization potential
Popliteal vein thrombosis	Nearly occlusive thrombus	Consistent with acute DVT
DVT extension	Distal into medial posterior tibial vein	Reflects propagation of DVT

**Legend:** RV—right ventricle; LV—left ventricle; RV/LV ratio—right-to-left ventricular diameter ratio; RA—right atrium; Vmax—maximum velocity; RV—RA gradient—right ventricle–right atrium pressure gradient; TAPSE—tricuspid annular plane systolic excursion; IVC—inferior vena cava; DVT—deep vein thrombosis.

## Data Availability

The original contributions presented in this study are included in the article/Supplementary Materials. Further inquiries can be directed to the corresponding authors.

## Supplementary Materials

The following supporting information can be downloaded at: https://www.mdpi.com/article/10.3390/diagnostics16010048/s1, 1. Cardiology Consultation in the Emergency Department; 2. CT Pulmonary Angiography findings; 3. Laboratory parameters collected in the Cardiology Department during hospitalization (Table S1); 4. Dynamic ultrasonographic evaluation in the Cardiology Unit prior to discharge.

## Abbreviations

The following abbreviations are used in this manuscript:

A A–a DO_2_	alveolar oxygen pressure alveolar–arterial oxygen gradient
a/A	arterial-to-alveolar oxygen ratio
ABG	arterial blood gas
ALP	alkaline phosphatase
ALT	alanine aminotransferase
AST	aspartate aminotransferase
aPTT	activated partial thromboplastin time
BE	base excess
BD	direct bilirubin
BP	blood pressure
bpm	beats per minute
CaO_2_	arterial oxygen content
CcO_2_	end-capillary oxygen content
CK	creatine kinase
CK-MB	creatine kinase MB isoenzyme
Cl	chloride
COHb	carboxyhemoglobin
CRP	C-reactive protein
CT	computed tomography
CTPA	computed tomography pulmonary angiography
DVT	deep vein thrombosis
ECG	electrocardiogram
ED	Emergency Department
eGFR	estimated glomerular filtration rate
ESR	erythrocyte sedimentation rate
EU	European Union
FA	folic acid
FiO_2_	fraction of inspired oxygen
GGT	gamma-glutamyl transferase
HBG	hemoglobin
HCO_3_^−^	bicarbonate ion
HCT	hematocrit
HDL	high-density lipoprotein cholesterol
HHb	deoxyhemoglobin
hs-CRP	high-sensitivity C-reactive protein
hs-cTnI	high-sensitivity cardiac troponin I
ICT	intracardiac thrombus
IMG	immature granulocytes
INR	international normalized ratio
IVC	inferior vena cava
K	potassium
LDH	lactate dehydrogenase
LDL	low-density lipoprotein cholesterol
LV	left ventricle
MCV	mean corpuscular volume
Mg	magnesium
MCH	mean corpuscular hemoglobin
MCHC mg mL mmHg µg	mean corpuscular hemoglobin concentration milligrams milliliters millimeters of mercury micrograms
MPV	mean platelet volume
Na ng	sodium nanograms
NT-proBNP O_2_	N-terminal pro–B-type natriuretic peptide oxygen
O_2_Cap	oxygen capacity
O_2_Ct	oxygen content
O_2_Hb	oxyhemoglobin
PCT	procalcitonin
PDW	platelet distribution width
PE	pulmonary embolism
PESI	Pulmonary Embolism Severity Index
pH	potential of hydrogen
PaCO_2_	arterial partial pressure of carbon dioxide
PaO_2_ pg	arterial partial pressure of oxygen picograms
PLT	platelets
POCUS	point-of-care ultrasound
POCUS-TTE	point-of-care transthoracic echocardiography
PSA	prostate-specific antigen
PT	prothrombin time
RA	right atrium
RBC	red blood cells
RV	right ventricle
RV/LV ratio	right-to-left ventricular diameter ratio
SaO_2_	arterial oxygen saturation
SO_2_	oxygen saturation
SpO_2_	peripheral oxygen saturation
TAPSE	tricuspid annular plane systolic excursion
TB	total bilirubin
TG	triglycerides
TR Vmax	tricuspid regurgitation maximum velocity
TSAT	transferrin saturation
TTE	transthoracic echocardiography
U.M.	unit of measure
USA Vitamin B12	United States of America cobalamin
Vmax	maximum velocity
VTE	venous thromboembolism
WBCs WI	white blood cells Wisconsin
