# Supplementary material for: Ultrasound Images That Speak: Assessing the Therapeutic Decision in the Emergency Department Regarding the Risk–Benefit Ratio of Systemic Thrombolysis in Intermediate-High-Risk Pulmonary Embolism—A Case Report
Source: Diagnostics (Basel). 2025 Dec 23;16(1):48. doi: 10.3390/diagnostics16010048 (PMC12786310; doi:10.3390/diagnostics16010048)
Supplement: Supplementary file 1 [file diagnostics-16-00048-s001.zip › diagnostics-4010275-supplementary.pdf]

diagnostics

Supplimentary Materials

1. Cardiology Consultation in the Emergency Department:

Blood pressure: 140/105 mmHg. **ECG:** Regular sinus rhythm, 100 bpm. Bifasic P wave in V1, S_1Q_3 pattern, P pulmonale in leads DII and DIII. Right ventricular overload with signs of acute strain and anterior T-wave inversions. **Laboratory findings:** Elevated cardiac biomarkers, hepatocytolysis, azotemia, hyperglycemia.

POCUS-TTE:
Pericardium normal (no effusion). Concentric left ventricular hypertrophy. Left ventricle not dilated (33 mm). Right ventricle markedly dilated (46 mm, measured below the pulmonary/tricuspid valve). Left atrium = 32 mm. Ascending aorta = 31 mm. Right atrium dilated. Pulmonary trunk dilated = 31 mm. Functional pulmonary regurgitation grade II/III. Color Doppler: Vmax = 3.2 m/s, maximum right ventricle–right atrium gradient = 40 mmHg. TAPSE = 12 mm. Mobile thrombus within the right atrium with tendency to prolapse through the tricuspid valve. IVC = 19 mm, inspiratory collapse < 50%. Congestive hepatomegaly with marked hepatic steatosis. Interventricular septum with paradoxical motion, "D-shaped" left ventricle.

Lower-limb venous ultrasound (left): Severe venous insufficiency with massive dilation of the deep femoral venous system (common femoral vein, superficial femoral vein) and saphenofemoral junction, with major incontinence of the great saphenous vein compared to the small saphenous vein.
Post-thrombotic changes (chronic sequelae) along the course of the small saphenous vein in the thigh.
Great saphenous vein, its junction, and superficial femoral vein compressible.
Nearly complete obstructive thrombosis of the popliteal vein, mildly heterogeneous echogenic appearance, extending distally into one of the posterior tibial veins (the medial one), while the other remains compressible. The proximal end of the deep venous thrombosis is located in the lower third of the superficial femoral vein, non-adherent to the wall, with an unstable pattern.
Recommendation: Pulmonary CT angiography – suspected pulmonary embolism with a massive thrombotic burden.

2. CT Pulmonary Angiography findings:

At the level of the right pulmonary artery, as well as at the level of the lobar, segmental, and subsegmental arteries supplying the upper, middle, and lower lobes, a nearly complete filling defect is visualized, consistent with acute pulmonary thromboembolism. No pulmonary infarction areas are identified. At the level of the left pulmonary artery, in its distal segment, as well as at the level of the lobar and segmental arteries of the lower lobe, the lobar artery supplying the lingula, and the lobar and segmental arteries of the anterior segment of the upper lobe, a partial filling defect is visualized, with the same significance of acute pulmonary thromboembolism. No pulmonary infarction areas are noted. A floating thrombus is visualized within the right atrium. Contrast reflux into the hepatic veins, consistent with right heart failure. No pulmonary parenchymal foci are identified. Main pulmonary artery: 32 mm; right pulmonary artery: 25 mm; left pulmonary artery: 23 mm. Mild interventricular septal

bowing toward the left. No pleural or pericardial effusion. Early spondylotic changes at the thoracic spine level. Within the scanned abdominal section: hepatic steatosis and a small hiatal hernia are noted. **Conclusion: Pulmonary thromboembolism with a massive bilateral thrombotic burden. Floating thrombus within the right atrium.**

3. Laboratory parameters collected in the Cardiology Department during hospitalization (**Table S1**)

Parameter Category	Test (U.M.)	Day 1	Day 2	Day 3	Day 4	Reference Range
**Hematology**	WBC (10^9/L)	8.68	6.89	6.06	5.73	4-10
	Neutrophils (10^9/L)	5.32	3.73	-	-	2000-7000
	Lymphocytes (10^9/L)	2.30	2.07	-	-	800-4000
	Monocytes (10^9/L)	0.98	0.94	-	-	120-1200
	Eosinophils (10^9/L)	0.04	0.12	-	-	20-500
	Basophils (10^9/L)	0.04	0.03	-	-	0 - 100
	RBC (10^{12}/L)	3.83	4.13	4.09	4.29	4.5-5.5
	HBG (g/dL)	13.5	14.4	14.3	14.9	13 -17
	HCT (%)	39.3	42.4	41.6	43.5	40 -54
	MCV (fL)	102.6	102.7	101.7	101.5	85 - 95
	MCHC (g/dL)	34.4	34.9	34.4	34.3	31-36
	PLT (10^9/L)	158	185	209	261	150 – 400
	MPV (fL)	12.2	11.8	11.5	11.6	7.2 – 11.7
	PDW (fL)	17.1	16.6	16.7	16.4	< 17
	Plateletcrit (%)	0.192	0.218	0.241	0.301	0.17 – 0.32
	ESR (mm/h)	15	-	-	-	< 20
**Coagulation**	aPTT (s)	47.6	-		-	23.5–36.5
	PT (s)	15.3	-	-	-	9–13
	Prothrombin index (%)	64	-	-	-	80–145 %
	INR	1.37	-	-	-	0.79 - 1.16
**Liver Function**	ALT (U/L)	64	-	-	-	< 50
	AST ((U/L)	81	-	-	-	< 50
	BD (mg/dL)	-	-	-	-	< 0.2
	TB (mg/dL)	-	-	-	-	0.3–1.2
	ALP (U/L)	-	49	-	-	30-120
	GGT (U/L)	-	151	-	-	<55
**Muscle Injury**	CK (U/L)	79	-	-	-	< 171
	CK-MB (U/L)	10.9	-	-	-	< 24
**Inflammation**	CRP mg/L	11.72	9.41	5.14	2.59	< 0.5
**Renal Function**	Creatinine (mg/dL)	1.5	-	0.94	0.68	0.67–1.17
	eGFR (mL/min/1.73 m^2)	47.82	-	84.15	80.01	> 60
	Urea (mg/dL)	92	-	49	41	17–43
**Glycemic Status**	Glucose (mg/dL)	86	-	-	-	74–106
**Electrolytes**	Na (mmol/L)	136	-	138	138	136–146

	K (mmol/L)	3.44	-	3.77	4.08	3.5–5.1
	Cl (mmol/L)	99	-	105	107	101–109
**Lipid profile**	Total Cholesterol (mg/dL)	172	-	-	-	< 200
	Cholesterol HDL (mg/dL)	44.4	-	-	-	40-55
	Cholesterol LDL (mg/dL)	105	-	-	-	< 129
	TG (mg/dL)	121	-	-	-	< 150
**Iron studies**	TSAT (%)	-	14	-	-	16-45
	Ferritin ng/mL	-	1192	-	-	20-250
	Serum Iron (µg/dL)	-	44	-	-	70-180
**Vitamin B12 and Folate panel**	Vitamin B12 (pg/mL)	-	239	-	-	180-914
	FA (ng/mL)	-	3.63	-	-	5.9-23.2
**Prostate tumor marker**	PSA (ng/ml)	-	0.58	-	-	< 4
**Sepsis biomarker**	PCT (ng/mL)	-	-	0.207	-	< 0.05

Legend: U.M. - unit of measurement; WBC – white blood cells; RBC – red blood cells; HBG – hemoglobin; HCT – hematocrit; MCV – mean corpuscular volume; MCHC – mean corpuscular hemoglobin concentration; PLT – platelets; MPV – mean platelet volume; PDW – platelet distribution width; ESR – erythrocyte sedimentation rate; aPTT – activated partial thromboplastin time; PT – prothrombin time; INR – international normalized ratio; ALT – alanine aminotransferase; AST – aspartate aminotransferase; BD – direct bilirubin; TB – total bilirubin; ALP – alkaline phosphatase; GGT – gamma-glutamyl transferase; CK – creatine kinase; CK-MB – creatine kinase MB isoenzyme; CRP – C-reactive protein; Creatinine – creatinine; eGFR – estimated glomerular filtration rate; Urea – urea; Glucose – glucose; Na – sodium; K – potassium; Cl – chloride; HDL cholesterol – high-density lipoprotein cholesterol; LDL cholesterol – low-density lipoprotein cholesterol; TG - triglycerides; TSAT – transferrin saturation; Vitamin B12 – cobalamin; FA – folic acid; PSA – prostate-specific antigen; PCT – procalcitonin.

4. Dynamic ultrasonographic evaluation in the Cardiology Unit prior to discharge:

Echocardiography: Normal-sized left ventricle. Mild concentric left ventricle hypertrophy, with preserved systolic function and grade I diastolic dysfunction. Normal-sized right ventricle, with preserved systolic function. No segmental wall-motion abnormalities. No signs of elevated pulmonary pressures (TR Vmax 2.7 m/s). Inferior vena cava with normal dimensions and respiratory variability. No pericardial effusion. No visible intracardiac thrombi.

Venous Doppler ultrasound of the lower limbs: Partial recanalization of the left popliteal vein; persistent non-compressible segment in the distal portion of the superficial femoral vein, with no flow detected, consistent with persistent thrombosis. On the right side, no ultrasound signs of thrombosis in the superficial femoral vein.
